# Variability of Hepatitis C Treatment Cascade Outcomes among People Who Inject Drugs across Geographically Diverse Clinics in the US: The HERO Study

**DOI:** 10.3390/v16101551

**Published:** 2024-09-30

**Authors:** Snehal S. Lopes, Moonseong Heo, Irene Pericot-Valverde, Brianna L. Norton, Lynn E. Taylor, Judith I. Tsui, Shruti H. Mehta, Judith Feinberg, Arthur Y. Kim, Paula J. Lum, Kimberly Page, Cristina Murray-Krezan, Jessica Anderson, Alain H. Litwin, the HERO Study Group

**Affiliations:** 1 Department of Public Health Sciences, Clemson University, Clemson, SC 29634, USA; 2 Department of Psychology, College of Behavioral, Social, and Health Sciences, Clemson University, Clemson, SC 29634, USA; 3 Albert Einstein College of Medicine, Montefiore Medical Center, Bronx, NY 10461, USA; 4 Department of Medicine, Montefiore Medical Center, Bronx, NY 10467, USA; 5 Department of Pharmacy Practice and Clinical Research, University of Rhode Island, 7 Greenhouse Road, Kingston, RI 02881, USA; 6 Department of Medicine, University of Washington, 325 9th Ave., Seattle, WA 98104, USA; 7 Department of Epidemiology, Johns Hopkins Bloomberg School of Public Health, 615 N. Wolfe Street, Room E6546, Baltimore, MD 21205, USA; 8 Department of Behavioral Medicine and Psychiatry, West Virginia University School of Medicine, 930 Chestnut Ridge Road, Morgantown, WV 26505, USA; 9 Department of Medicine, Section of Infectious Diseases, West Virginia University School of Medicine, 1 Medical Center Drive, Morgantown, WV 26506, USA; 10 Division of Infectious Diseases, Massachusetts General Hospital, 55 Fruit St., Boston, MA 02114, USA; 11 Infectious Disease, Mass General Research Institute, Harvard Medical School, Boston, MA 02115, USA; 12 Department of Medicine, University of California, 1001 Potrero Ave., San Francisco, CA 94110, USA; 13 Department of Internal Medicine, University of New Mexico Health Sciences Center, University of New Mexico, MSC 10 5550, Albuquerque, NM 87131, USA; 14 Department of Medicine, University of Pittsburgh, Pittsburgh, PA 15215, USA; 15 School of Health Research, Clemson University, Clemson, SC 29634, USA; 16 Department of Medicine, University of South Carolina School of Medicine, 876 W Faris Rd., Greenville, SC 29605, USA; 17 Department of Medicine, Prisma Health, Greenville, SC 29605, USA

**Keywords:** clinical site, HCV treatment cascade outcomes, PWID, intraclass correlation coefficient, cluster randomized trials

## Abstract

Heterogeneity of outcomes across different clinical trial study sites is often inevitable. Understanding how outcomes differ by site is important for planning future programs and studies. We examined the extent of heterogeneity of hepatitis C virus (HCV) treatment cascade outcomes among persons who inject drugs (PWIDs) across sixteen clinical sites utilized in the HERO Study—a pragmatic randomized trial of HCV treatment support. Treatment cascade outcomes included averages of overall treatment adherence and proportions of treatment initiation, treatment completion, sustained virologic response (SVR) test completion, and SVR achievement. The HERO study utilized 16 clinical sites across the United States (US): eight opioid treatment programs (OTPs) and eight community health centers (CHCs). Variability of the outcomes across the 16 clinical sites was assessed using ranges and intraclass correlation coefficients (ICC) estimated from mixed-effects linear or logistic regression models. Treatment initiation was analyzed in the intention-to-treat (ITT) sample (N = 755); treatment completion, adherence, and SVR test completion in the modified ITT (mITT) sample, which is the sample that initiated treatment (N = 623); and SVR achievement in the mITT and per-protocol (PP, N = 501) samples. Across the 16 clinical sites, the range observed in the averages of overall treatment adherence was from 68% to 81% [ICC = 0.026 (0.005, 0.054)], and the ranges of proportions observed were from 68% to 96% for treatment initiation [ICC (95% CI) = 0.086 (0.051, 0.155)], 60% to 100% for treatment completion [ICC = 0.049 (0.008, 0.215)], 54% to 95% for SVR test completion [ICC = 0.096 (0.006, 0.177)], 46% to 90% for SVR achievement in the mITT sample [ICC = 0.070 (0.014, 0.122)], and 76% to 100% for SVR achievement in the PP sample [ICC = 0.143 (0.021, 0.422)]. The variability of the outcomes across 16 US sites treating HCV among PWIDs appears to be substantial in view of the ranges and ICC values of the outcomes. It is imperative to develop tailored interventions to target the sources of variability and reduce barriers at the patient, provider, clinic, and state policy levels to facilitate more equitable access to HCV treatment and reduce heterogeneity in treatment outcomes.

## 1. Introduction

New emerging advancements in Hepatitis C virus (HCV) treatment underscore the need for well-designed clinical trials to build a strong evidence base about the efficacy and effectiveness of the treatments among persons who inject drugs (PWIDs) while reducing biases in analyzing the treatment effects. Many large randomized controlled trial (RCT) studies are conducted across multiple sites to improve generalizability of the study findings, increase participant recruitment rates, and increase statistical power when event rates in the outcome are rare [1]. Cluster randomized controlled trial (cRCT) studies are often designed, and therefore, there is a need for cRCT studies to assess the effects of clustering during the planning stages as well as account for the clustering effects in the analyses of the trial outcomes [2,3]. 

A few studies have focused on variability in treatment cascade outcomes stemming from environmental factors such as the clinical site, especially in the direct-acting antiviral (DAA) treatment era. In the DAA era, there are some studies exploring variability in treatment initiation by clinical site [4,5]. For instance, the study by Saeed et al. (2017) among persons with HIV-HCV coinfection in Canada found differences in treatment initiation by province [4]. With respect to the outcome of treatment completion, a prior study in Australia in the general HCV population found significant variability across states [6]. In the US, some studies have assessed variance in treatment outcomes across sites within a single state or single center in the general HCV population, but interferon-based treatments and DAA treatments were not analyzed separately [7,8]. To our knowledge, few studies have explored variability in DAA treatment adherence by clinical site in the general HCV or PWID population. Overall, there is a lack of studies assessing the variabilities of HCV DAA treatment-related outcomes in the US, especially in the PWID population. 

We aimed to assess the variability of the whole spectrum of HCV DAA treatment-related cascade outcomes, including treatment initiation, completion, adherence, SVR test completion, and SVR achievement across clinical sites using data from a large multisite clinical trial study conducted among PWIDs with HCV infection in the US. 

## 2. Materials and Methods

### 2.1. The Study Design and Population

This was a secondary data analysis study using data from the Hepatitis C Real Option (HERO) study (ClinicalTrials.gov, NCT02824640) [9,10]. The HERO study randomized participants at the individual participant level to one of two HCV treatment models: (i) modified Directly Observed Treatment (mDOT) or (ii) Patient Navigation (PN). The study population included PWIDs with recent drug use history (defined as self-reporting active substance injection use within 90 days of screening) and current HCV infection. As part of the study procedures, all participants received an oral fixed-dose combination tablet of sofosbuvir (400 mg) with velpatasvir (100 mg) to be taken once daily for 12 weeks. The medications were provided by the study, and the medication treatment regimen was identical across the study sites. Three sample types were defined as follows: (i) the intention-to-treat (ITT) sample included all participants who were randomized to either of the two study treatment arms (N = 755), (ii) the modified intention-to-treat (mITT) sample included participants who were randomized to the study treatment arms and initiated treatment (N = 623), and (iii) the per-protocol (PP) sample included participants who received the study intervention as assigned to them through the randomization process and had a determined SVR status after the end of the treatment (N = 501). The study compared the two HCV treatment models with respect to the outcomes of treatment initiation, completion, DAA adherence, and HCV cure and found no significant differences. Details of the HERO Study and primary outcomes have been published elsewhere [9,10].

### 2.2. Measures

#### 2.2.1. Clinical Sites

The HERO study recruited participants from 23 clinical sites spread across eight cities in eight states (California, Massachusetts, Maryland, New Mexico, New York, Rhode Island, Washington, and West Virginia) in the US. The 23 clinical sites consisted of 15 community health centers (CHCs) and eight opioid treatment programs (OTPs). That is, each city utilized one OTP site and one or more CHC site, depending on availability. However, for cities with multiple CHCs, each CHC site was not separately identified or coded; for this reason, multiple CHC clinics within a city were coded as a single city-level CHC site; that is, the 15 CHC sites were collapsed into eight CHC sites (corresponding to each of the eight cities) in our analyses. The Supplemental Files, Tables S1 and S2, provide information on the baseline characteristics of the study population at the eight OTP sites and eight CHC sites, respectively.

#### 2.2.2. Treatment Cascade Outcomes

The HCV treatment cascade outcomes were as follows:

1. Treatment initiation: This was a binary variable (yes/no) indicating whether participants initiated the HCV DAA treatment.
2. Treatment completion: This was a binary variable (yes/no) indicating whether the participants completed the HCV DAA treatment, with treatment completion defined as being retained in treatment for ≥84 days.
3. Overall adherence to HCV DAAs: Adherence to DAA treatment was measured using electronic blister packs, which recorded the time of medication removal. Overall adherence was calculated as an average of any available/non-missing weekly adherence rate over the 12-week treatment period.
4. SVR test completion: This was a binary variable (yes/no) indicating whether the participants were available for SVR testing.
5. SVR achievement: This was a binary variable (yes/no) indicating whether the participants achieved SVR. SVR achievement was defined as having an HCV RNA level below the quantitation limit (≤15 IU/mL) and was determined to be between 70 and 365 days after the end of the treatment.

### 2.3. Statistical Analyses

Descriptive statistics were computed for each of the treatment cascade outcomes by the 16 clinical sites. The between-site correlation was assessed using the intraclass correlation coefficient (ICC), which measures the within-cluster relatedness of responses as the proportion of total outcome variance explained by between-site outcome variance [11] using variance component estimates from mixed-effects linear regression for the continuous outcome of treatment adherence and mixed-effects logistic regression models for the binary outcomes. The mixed-effects regression analyses included the intercept as site-specific random effects. In the mixed-effect logistic regression model that we employed, the within-cluster variation is quantified by π^2^/3 (which is the variance of a standard logistic distribution), the between-cluster variation is quantified by the variance of the site-specific random intercept term, and the total variation is the sum of the within- and between-cluster variations. If the ICC = between-cluster variation/(between-cluster variation + within-cluster variation) is higher, then the within-cluster relatedness of responses, or equivalent homogeneity of outcomes across subjects within a cluster is relatively large such that outcomes among within clusters are more homogenous than when the ICC is lower in which within-cluster variation is larger, yielding more heterogeneous outcomes within clusters. Bootstrap 95% confidence intervals (95% CI) were computed for the ICC estimates. Treatment initiation was analyzed in the ITT sample. Treatment adherence, completion, and SVR test completion were analyzed in the mITT sample. The SVR test result was analyzed in the mITT and PP samples. All analyses were conducted using SAS 9.4. 

## 3. Results

**Figure 1:**
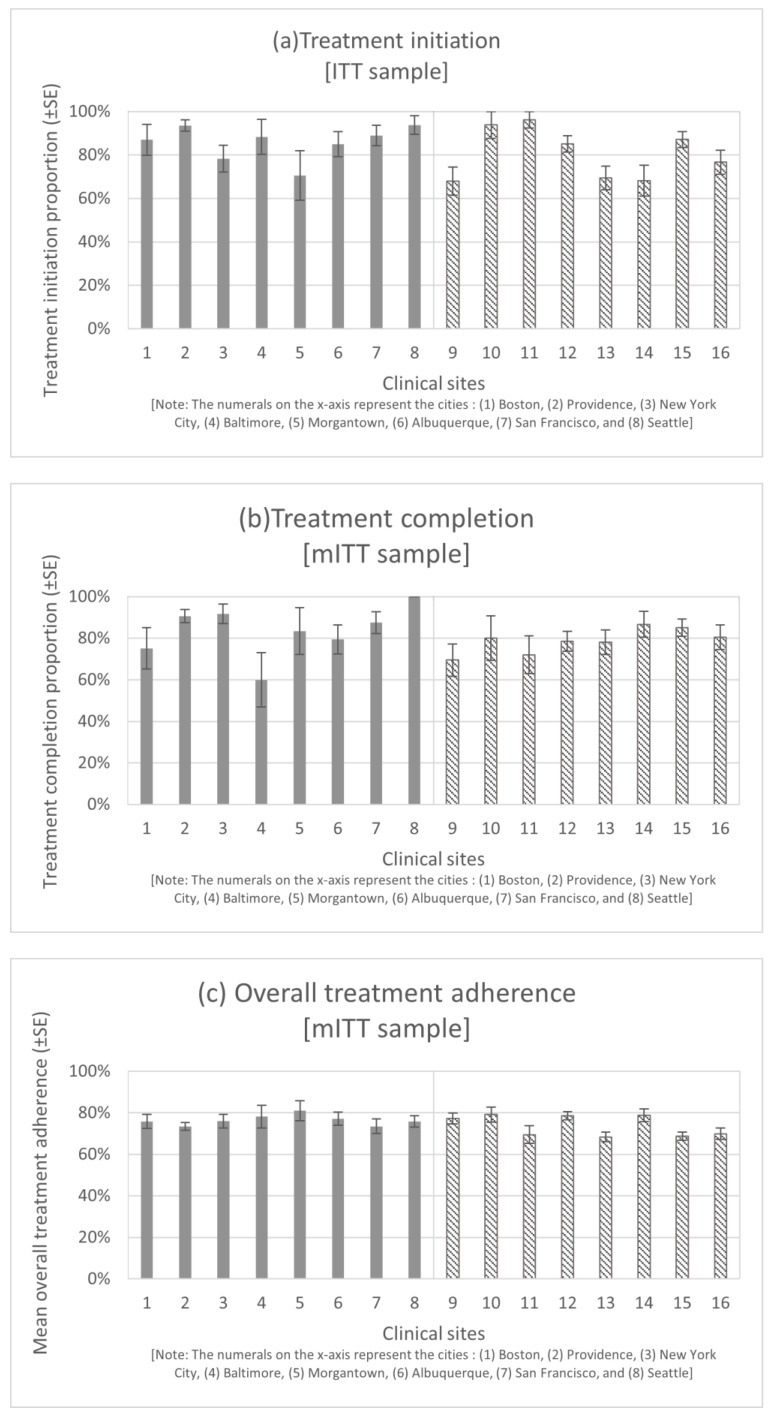

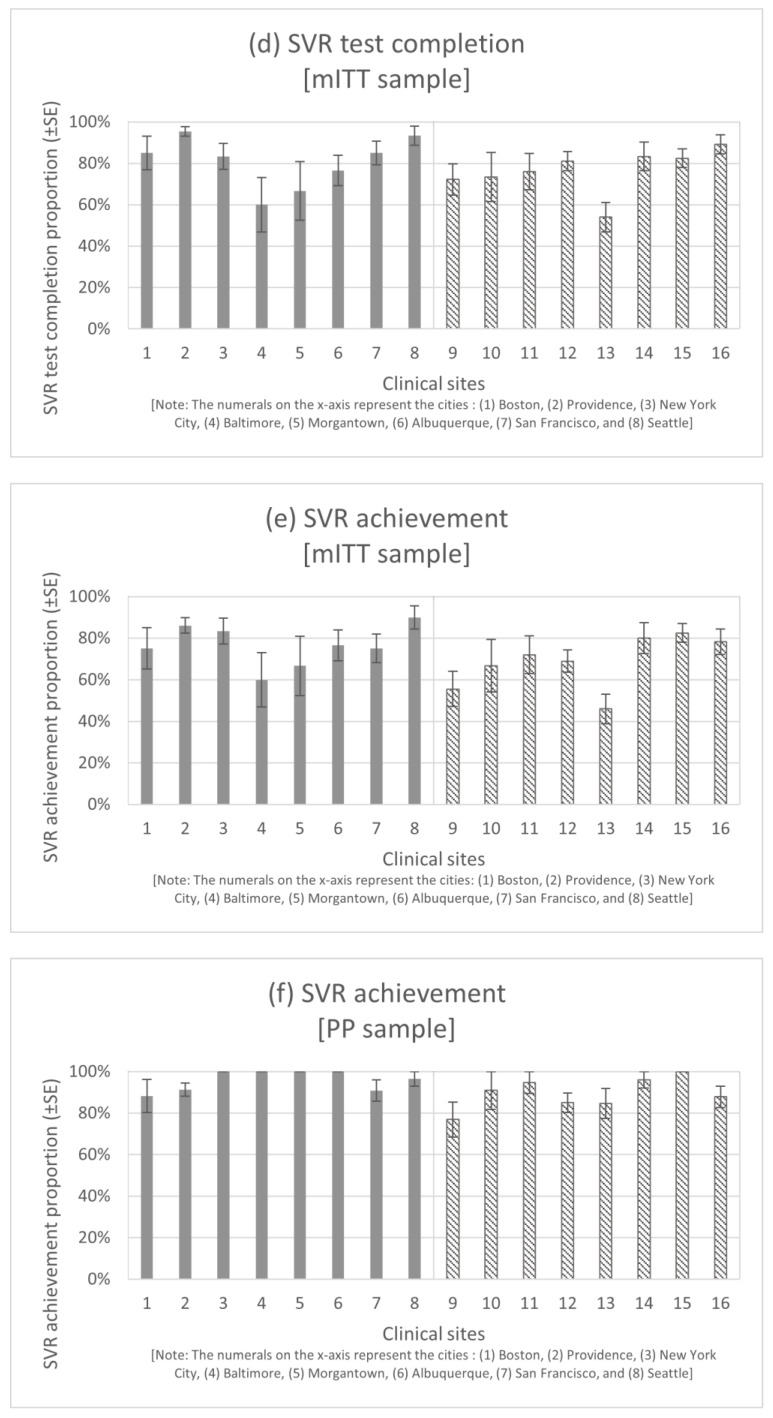
Distribution of the study outcomes by clinical sites. The bars in the charts indicate the outcomes within each of the 16 clinical sites. The error bars are for standard errors (SE). The numerals on the *x*-axis represent the cities where the OTP/CHC sites were located as follows: (1) Boston, (2) Providence, (3) New York City, (4) Baltimore, (5) Morgantown, (6) Albuquerque, (7) San Francisco, and (8) Seattle. Grey and striped bars have been used to distinguish between the OTP and CHC sites. Abbreviations: intention-to-treat (ITT), modified intention-to-treat (mITT), per-protocol (PP), opioid treatment program (OTP), community health center (CHC); sustained virologic response (SVR).

Table 1 shows the ICC values and outcome ranges across the 16 clinical sites and across OTP sites and CHC sites separately. The outcomes for each of the clinical sites are presented in Figure 1. 

### 3.1. Treatment Initiation

The treatment initiation proportion was 94% at the maximum and 71% at the minimum among the OTP sites, whereas the treatment initiation proportion at the maximum was 96% and, at the minimum, was 68% among the CHC sites. The ICC for treatment initiation was estimated to be 0.086 (95% CI: 0.051, 0.155) among the 16 clinical sites.

### 3.2. Treatment Completion

Among the OTP sites, the treatment completion proportion at its highest was 100% and at its lowest was 60%. Among the CHC sites, the treatment completion proportions were 87% and 69% at their highest and lowest, respectively. The ICC for treatment completion was estimated to be 0.049 (0.008, 0.215) among the 16 clinical sites.

### 3.3. Overall Treatment Adherence

The average overall treatment adherence reached a maximum of 81% and a minimum of 73% among the OTP sites, whereas among the CHC sites, the maximum and minimum averages were 79% and 68%, respectively. The ICC was estimated to be 0.026 (0.005, 0.054) for overall treatment adherence among the 16 clinical sites.

### 3.4. SVR Test Completion

SVR test completion proportions were 95% at the highest and, 60% at the lowest among the OTP sites, and 89% at the highest and 54% at the lowest among the CHC sites. The ICC for SVR test completion was estimated to be 0.096 (0.006, 0.177) among the 16 clinical sites.

### 3.5. SVR Achievement

In the mITT sample, the highest and lowest SVR achievement proportions were 90% and 60%, respectively, among the OTP sites, and 82% and 46%, respectively, among the CHC sites. In the PP sample, the highest SVR achievement proportion of 100% was achieved at four locations among the OTP sites and at one location among the CHC sites. The lowest SVR achievement proportion was 88% among the OTP sites. Among the CHC sites, the highest and lowest SVR achievement proportions were 100% and 77%, respectively. The ICC for SVR achievement was estimated to be 0.070 (0.014, 0.122) in the mITT sample and 0.143 (0.021, 0.422) in the PP sample among the 16 clinical sites.

## 4. Discussion

The results of our analyses found that based on the ICC values across the 16 clinical sites, less than 10% of the variance in each of the study outcomes was attributable to clinic site level outcome variability, with the exception of SVR in the PP sample analyses where the variance attributable to clinical sites was estimated to be 14%. However, the extent of variance attributable to clinical sites in our study is still relatively high, considering the norm for human studies (between 1% and 2%) [11]. There was a difference of 13% between the maximum and minimum average overall treatment adherence across clinical sites. The difference between the maximum and minimum proportions of the study outcomes across clinical sites was as much as 28% for treatment initiation, 40% for treatment completion, 41% for SVR test completion, 44% and 23% for SVR achievement in the mITT and PP samples, respectively. Overall, the variability in terms of ICC appears to be substantial compared to studies in other research areas; for instance, the median (IQR) of ICCs among 246 school-based studies was 0.03 (0.01, 0.08) [12]. Therefore, when designing cRCT studies among the PWID population, the cluster effect size in terms of ICC should not be assumed low at below 0.05, which might yield smaller sample size determinations and thus inadequate power. However, based on our estimations, ICC should be conservatively assumed to be between 0.1 and 0.15 as a lower bound for designing and conducting sufficiently powered cluster randomized studies to detect significant intervention or treatment effects. Disregarding such potential clustering effects could lead to biased treatment effect estimates, underestimation of the uncertainty, and lower *p*-values [13].

In the DAA era, treatment initiation has varied by clinical site in several prior studies [4,5]. Among persons with HIV-HCV coinfection in Canada, the maximum difference in the likelihood of treatment initiation between the provinces in a prior study was 4% [4] and between centers in another study was 220% [5]. In an Australian study, significant variations in treatment completion outcomes were observed across states, ranging from 24% lower to 32% higher compared to the reference state [6]. Also, the prior study found differences in treatment completion by region defined based on the level of remoteness, where the likelihood of treatment completion was 48% lower for the remotest of regions as compared to major city regions [6]. To our knowledge, few studies have explored variability in HCV DAA treatment adherence and SVR by clinical site in the general HCV or PWID population. Our study contributes to the understanding about the variability in treatment outcomes specifically for the PWID population across clinics in multiple cities in the US.

Our study also assessed the variability of the treatment cascade outcomes across OTP sites and CHC sites separately. Based on ICC values, the variability attributable to clinical sites exceeded 10% for the outcomes of treatment completion and SVR test completion among the OTP sites, and for the outcome of SVR achievement (in the PP sample) among the CHC sites. Except for treatment completion, the differences in the minimum and maximum values of the other outcomes were greater among the CHC sites than among the OTP sites. Greater variability in the outcomes among the CHC sites could be due to the broader health focus of CHC clinics and, therefore, greater variety of the services and practices at CHCs.

There could be several reasons to explain why variabilities in HCV treatment outcomes exist by clinical sites, including environmental factors such as educational facilities, living conditions, level of neighborhood segregation (the major mechanisms resulting in neighborhood segregation in the US have been race and economic status [14]), rural/metropolitan status of residential area [6], as well as healthcare policies [5,15]. Health policies vary across states, and even within states, there can be regional variations in payer policies, which can cause variations in treatment uptake [5,15]. The high cost of DAA treatment has led states and insurance companies to resort to cost-cutting strategies such as laying strict restrictions on reimbursement for treatment based on criteria such as patient behavioral characteristics (e.g., substance use), physical conditions (e.g., stage of liver fibrosis), the provider type (e.g., specialists), and health insurance type (e.g., not covered by Medicaid) [5,7]. Adverse environmental factors make differential access to healthcare a systemic problem. Besides the environmental factors, differences in the demographic and clinical profiles of the patient population served by the clinics (such as age and diagnosis of cirrhosis) may also cause variations in HCV treatment outcomes [16].

The need to ensure equity in health makes it all the more essential that clinical trials are inclusive of the diverse health experiences within the target population so as to inform treatment response, decision-making, and patient counseling in healthcare settings and motivate further research on the development of treatments that are effective as well as equitable with respect to their health benefits [17]. While variability attributable to many of the factors may be beyond the control of the study, it is essential from an implementation science point of view that efforts are made to reduce the variability in HCV treatment outcomes arising from factors related to the study (such as having a multi-site design) as much as possible by tailoring HCV treatment delivery protocols to address site-specific disadvantages that affect access to treatment. For example, some clinical settings may have lower treatment initiation proportions than others due to differences in procedures for linking patients to care [8], in which case clinical trial studies may need to incorporate additional measures in the treatment delivery process at such sites to enhance care coordination and linkage to HCV care.

Our study has some limitations that should be acknowledged. First, our results may be limited due to the lack of clinic-level characteristics that could explain the noted outcome heterogeneities, and due to the clinic sites not being randomly selected. Second, since the HERO study was a pragmatic trial, there may have been some heterogeneity in the delivery of the interventions across the different sites, which may have contributed to some of the variability in the outcomes [9,10]. Third, since there was no identifier assigned during the study period to differentiate between individual CHCs from the same city, we analyzed CHCs from the same city as a single cluster. We determined that collapsing CHCs from the same city into a single cluster was an appropriate solution, as CHCs located in the same city could have similar environmental influences [18]. However, combining CHCs from the same city into a single cluster makes our analysis more of a “site/city” CHC level analysis than a “CHC clinic” level analysis. Fourth, all outcomes other than treatment initiation were analyzed only among those who initiated treatment. It is possible that the participants who did not initiate treatment may be different from those who initiated treatment, which could have introduced some bias in the ICC estimates provided by our study. Fifth, the HERO study was not a cRCT study which reduces the utility of our results when planning another cRCT. However, while it would be ideal to estimate ICCs only based on cRCTs, the use of ICC estimates from cRCTs available in the literature for designing another cRCT may not necessarily be appropriate if the contexts of the prior cRCT studies are different [19]. In the absence of any prior cRCTs reporting ICCs to provide an estimate of variability between clinical sites in the specific context of HCV DAA treatment among PWIDs, we believe that the ICC estimates from our study still hold value as they could guide study investigators designing future cRCTs for HCV treatment outcomes. That is, although the strength of the within-site correlation may be lesser in a multisite individually randomized trial as compared to a cRCT, the ICC values from the HERO multisite trial could be used as a reasonable lower bound estimate of ICCs for designing a cRCT focusing on HCV DAA treatment among PWIDs for appropriate power analysis and sample size determinations. (Note: Although the HERO study was not a cRCT, the ICC is still an appropriate metric to estimate the clustering effect of sites or within-site correlations of outcomes in multisite individually randomized trials as well because treatment outcomes for individuals within the same clinical site could be correlated due to similarity of environmental experiences or other factors [12].) Finally, it is possible that the patient navigators formed another layer of clustering within sites. However, the navigators did not necessarily work with a fixed set of participants, and the number of navigators was limited at each site. Therefore, it was not possible to assess their potential clustering effects.

## 5. Conclusions

In conclusion, our study adds evidence to the scientific literature about the extent of variability in HCV DAA treatment cascade outcomes across clinical sites that are widely dispersed geographically using a large dataset among PWIDs with HCV infection in the US. Our findings also highlight the need for the development of HCV treatment delivery protocols that are tailored to address specific barriers at multiple levels, including patient, provider, clinic, payer, and state policy levels, to ensure more equitable access to HCV treatment and consequently, minimize heterogeneity of HCV treatment outcomes.

## Figures and Tables

**Table 1:** Treatment-related cascade outcome ranges and ICC values across the clinical sites.

		All 16 Sites		8 OTP Sites		8 CHC Sites	
HCV Treatment Cascade Outcome	Study Sample	Range of Outcome [Minimum–Maximum]	ICC (95% CI)	Range of Outcome [Minimum–Maximum]	ICC (95% CI)	Range of Outcome [Minimum–Maximum]	ICC (95% CI)
Treatment initiation (%)	ITT	68–96%	0.086 (0.051, 0.155)	71–94%	0.049 (0.004, 0.143)	68–96%	0.090 (0.040, 0.244)
Treatment completion (%)	mITT	60–100%	0.049 (0.008, 0.215)	60–100%	0.138 (0.018, 0.490)	69–87%	not estimable ^a^
Overall treatment adherence (mean)	mITT	68–81%	0.026 (0.005, 0.054)	73–81%	not estimable ^a^	68–79%	0.059 (0.035, 0.098)
SVR test completion (%)	mITT	54–95%	0.096 (0.006, 0.177)	60–95%	0.128 (0.012, 0.272)	54–89%	0.069 (0.004, 0.162)
SVR achievement (%)	mITT	46–90%	0.070 (0.014, 0.122)	60–90%	0.023 (0.004, 0.116)	46–82%	0.076 (0.008, 0.145)
	PP	77–100%	0.143 (0.021, 0.422)	88–100%	0.073 (0.014, 0.571)	77–100%	0.163 (0.013, 0.563)

Note: ^a^ Not estimable as the estimated G matrix was not positive definite. Abbreviations: intra-cluster correlation coefficient (ICC), confidence interval (CI), sustained virologic response (SVR), intention-to-treat (ITT), modified intention-to-treat (mITT), per-protocol (PP), opioid treatment program (OTP), community health center (CHC). The treatment adherence outcome is based on non-missing adherence data only.

## Data Availability

The dataset used for the current study is not publicly available because it contains information that could compromise the privacy of the research participants. The dataset is available from the corresponding author upon reasonable request.

## Supplementary Materials

The following supporting information can be downloaded at: https://www.mdpi.com/article/10.3390/v16101551/s1, Table S1: Baseline characteristics of the study participants from OTP sites; Table S2: Baseline characteristics of the study participants from CHC sites.
