# Supplementary material for: Variability of Hepatitis C Treatment Cascade Outcomes among People Who Inject Drugs across Geographically Diverse Clinics in the US: The HERO Study
Source: Viruses. 2024 Sep 30;16(10):1551. doi: 10.3390/v16101551 (PMC11512280; doi:10.3390/v16101551)
Supplement: Supplementary file 1 [file viruses-16-01551-s001.zip › viruses-3087082-supplementary.pdf]

Supplementary Table S1

Characteristic	Total (N=312)	OTP 1 (N=23)	OTP 2 (N=92)	OTP 3 (N=46)	OTP 4 (N=17)	OTP 5 (N=17)	OTP 6 (N=40)	OTP 7 (N=45)	OTP 8 (N=32)	p
**Baseline Characteristics of the Study Participants From OTP Sites**										
**Gender**										0.212
Female	98	6 (6.1%)	31 (31.6%)	11 (11.2%)	6 (6.1%)	8 (8.2%)	15 (15.3%)	7 (7.1%)	14 (14.3%)	
Male	210	17 (8.1%)	60 (28.6%)	35 (16.7%)	11 (5.2%)	9 (4.3%)	25 (11.9%)	36 (17.1%)	17 (8.1%)	
Transgender or Gender Non-conforming	4	0 (0.0%)	1 (25.0%)	0 (0.0%)	0 (0.0%)	0 (0.0%)	0 (0.0%)	2 (50.0%)	1 (25.0%)	
**Age [M (SD)]**	42.4 (11.2)	45.3 (11.0)	37.7 (8.8)	43.7 (11.0)	46.2 (14.6)	35.2 (7.6)	39.9 (11.9)	49.2 (10.3)	47.0 (10.1)	<0.001
**Race**										<0.001
White/Caucasian	197	18 (9.1%)	71 (36.0%)	5 (2.5%)	10 (5.1%)	17 (8.6%)	24 (12.2%)	26 (13.2%)	26 (13.2%)	
Black/African American	25	3 (12.0%)	1 (4.0%)	6 (24.0%)	5 (20.0%)	0 (0.0%)	1 (4.0%)	8 (32.0%)	1 (4.0%)	
Other	74	2 (2.7%)	20 (27.0%)	34 (45.9%)	2 (2.7%)	0 (0.0%)	0 (0.0%)	11 (14.9%)	5 (6.8%)	
**Latino/Hispanic Ethnicity**										<0.001
No	223	21 (9.4%)	76 (34.1%)	12 (5.4%)	17 (7.6%)	17 (7.6%)	10 (4.5%)	40 (17.9%)	30 (13.5%)	
Yes	89	2 (2.2%)	16 (18.0%)	34 (38.2%)	0 (0.0%)	0 (0.0%)	30 (33.7%)	5 (5.6%)	2 (2.2%)	
**Marital/cohabitation Status**										0.009
Single/Separated /Divorced/Widowed	260	22 (8.5%)	84 (32.3%)	34 (13.1%)	10 (3.8%)	13 (5.0%)	32 (12.3%)	37 (14.2%)	28 (10.8%)	
Married/living together as married	46	0 (0.0%)	7 (15.2%)	9 (19.6%)	6 (13.0%)	4 (8.7%)	8 (17.4%)	8 (17.4%)	4 (8.7%)	
Other	3	0 (0.0%)	0 (0.0%)	2 (66.7%)	1 (33.3%)	0 (0.0%)	0 (0.0%)	0 (0.0%)	0 (0.0%)	
**Education**										<0.001
Less than HS	82	0 (0.0%)	21 (25.6%)	21 (25.6%)	6 (7.3%)	1 (1.2%)	13 (15.9%)	14 (17.1%)	6 (7.3%)	
HS diploma or GED	118	12 (10.2%)	33 (28.0%)	20 (16.9%)	3 (2.5%)	8 (6.8%)	18 (15.3%)	14 (11.9%)	10 (8.5%)	
Some college or more	109	10 (9.2%)	37 (33.9%)	4 (3.7%)	8 (7.3%)	8 (7.3%)	9 (8.3%)	17 (15.6%)	16 (14.7%)	
**Living stability**										0.684
Stable housing	134	11 (8.2%)	37 (27.6%)	20 (14.9%)	7 (5.2%)	11 (8.2%)	15 (11.2%)	18 (13.4%)	15 (11.2%)	
Unstable housing	175	11 (6.3%)	54 (30.9%)	25 (14.3%)	10 (5.7%)	6 (3.4%)	25 (14.3%)	27 (15.4%)	17 (9.7%)	
**Availability of transportation**										<0.001
Yes	129	14 (10.9%)	53 (41.1%)	3 (2.3%)	4 (3.1%)	15 (11.6%)	16 (12.4%)	21 (16.3%)	3 (2.3%)	
Maybe, if I can get a ride	23	0 (0.0%)	10 (43.5%)	0 (0.0%)	0 (0.0%)	2 (8.7%)	10 (43.5%)	0 (0.0%)	1 (4.3%)	
Maybe, if public transportation is available	151	8 (5.3%)	23 (15.2%)	41 (27.2%)	13 (8.6%)	0 (0.0%)	14 (9.3%)	24 (15.9%)	28 (18.5%)	
No	5	0 (0.0%)	4 (80.0%)	1 (20.0%)	0 (0.0%)	0 (0.0%)	0 (0.0%)	0 (0.0%)	0 (0.0%)	
**Employed with a regular job or informal work**										0.004
Yes	116	8 (6.9%)	44 (37.9%)	11 (9.5%)	3 (2.6%)	12 (10.3%)	15 (12.9%)	13 (11.2%)	10 (8.6%)	
No	192	13 (6.8%)	47 (24.5%)	34 (17.7%)	14 (7.3%)	5 (2.6%)	25 (13.0%)	32 (16.7%)	22 (11.5%)	
**Medication for OUD**										<0.001
None	18	1 (5.6%)	0 (0.0%)	13 (72.2%)	0 (0.0%)	0 (0.0%)	2 (11.1%)	1 (5.6%)	1 (5.6%)	
Buprenorphine only	5	0 (0.0%)	0 (0.0%)	0 (0.0%)	0 (0.0%)	0 (0.0%)	4 (80.0%)	0 (0.0%)	1 (20.0%)	
Methadone	276	20 (7.2%)	92 (33.3%)	30 (10.9%)	17 (6.2%)	17 (6.2%)	30 (10.9%)	40 (14.5%)	30 (10.9%)	
**Depressive symptoms (PHQ-9) [M (SD)]**	9.9 (6.6)	10.3 (7.5)	10.7 (6.9)	8.1 (7.3)	8.8 (4.6)	8.8 (5.7)	10.6 (6.3)	9.3 (5.8)	10.7 (6.9)	0.461
**Anxiety symptoms (GAD-7) [M (SD)]**	8.2 (5.9)	7.8 (6.4)	9.3 (5.9)	6.7 (6.5)	7.7 (5.0)	6.3 (4.6)	8.6 (5.5)	7.5 (5.3)	9.2 (6.6)	0.163
**Previously Received HCV Treatment (non-DAA)**										0.389
No	293	20 (6.8%)	84 (28.7%)	42 (14.3%)	17 (5.8%)	17 (5.8%)	39 (13.3%)	43 (14.7%)	31 (10.6%)	
Yes	19	3 (15.8%)	8 (42.1%)	4 (21.1%)	0 (0.0%)	0 (0.0%)	1 (5.3%)	2 (10.5%)	1 (5.3%)	

**Cirrhosis**										0.010
No	295	19 (6.4%)	90 (30.5%)	42 (14.2%)	14 (4.7%)	16 (5.4%)	40 (13.6%)	42 (14.2%)	32 (10.8%)	
Yes	17	4 (23.5%)	2 (11.8%)	4 (23.5%)	3 (17.6%)	1 (5.9%)	0 (0.0%)	3 (17.6%)	0 (0.0%)	
**HIV infection (positive)**										<0.001
No	182	18 (9.9%)	60 (33.0%)	28 (15.4%)	5 (2.7%)	10 (5.5%)	30 (16.5%)	0 (0.0%)	31 (17.0%)	
Yes	13	1 (7.7%)	2 (15.4%)	4 (30.8%)	0 (0.0%)	0 (0.0%)	0 (0.0%)	6 (46.2%)	0 (0.0%)	
**Alcohol misuse**										0.080
No	222	16 (7.2%)	74 (33.3%)	31 (14.0%)	11 (5.0%)	9 (4.1%)	30 (13.5%)	25 (11.3%)	26 (11.7%)	
Yes	73	5 (6.8%)	17 (23.3%)	12 (16.4%)	6 (8.2%)	8 (11.0%)	6 (8.2%)	14 (19.2%)	5 (6.8%)	
**Last drug injection (within 3 months of screening)**										0.100
0-4 weeks	235	17 (7.2%)	62 (26.4%)	38 (16.2%)	11 (4.7%)	10 (4.3%)	34 (14.5%)	41 (17.4%)	22 (9.4%)	
5-8 weeks	48	3 (6.3%)	20 (41.7%)	6 (12.5%)	4 (8.3%)	4 (8.3%)	2 (4.2%)	4 (8.3%)	5 (10.4%)	
9-12 weeks	29	3 (10.3%)	10 (34.5%)	2 (6.9%)	2 (6.9%)	3 (10.3%)	4 (13.8%)	0 (0.0%)	5 (17.2%)	
**Number of days injected drugs in the past 3 months [M (SD)]**	30.9 (30.3)	27.3 (34.4)	31.3 (30.8)	36.2 (33.4)	36.1 (34.6)	34.9 (24.4)	28.3 (27.1)	35.9 (29.2)	16.0 (23.7)	0.028
**Times injecting drugs a day [M (SD)]**	2.8 (2.8)	4.0 (6.4)	2.9 (3.0)	3.3 (2.6)	2.1 (1.6)	3.2 (2.8)	2.7 (1.9)	2.3 (1.4)	1.8 (1.0)	0.129
**Urine drug screen results at baseline visit :**										
Any drug										
No	2	1 (50.0%)	0 (0.0%)	0 (0.0%)	0 (0.0%)	0 (0.0%)	1 (50.0%)	0 (0.0%)	0 (0.0%)	0.200
Yes	284	18 (6.3%)	91 (32.0%)	40 (14.1%)	16 (5.6%)	16 (5.6%)	33 (11.6%)	41 (14.4%)	29 (10.2%)	
Amphetamine										
Positive	63	0 (0.0%)	8 (12.7%)	0 (0.0%)	0 (0.0%)	3 (4.8%)	16 (25.4%)	23 (36.5%)	13 (20.6%)	<0.001
Negative	223	19 (8.5%)	83 (37.2%)	40 (17.9%)	16 (7.2%)	13 (5.8%)	18 (8.1%)	18 (8.1%)	16 (7.2%)	
Methamphetamine										
Positive	77	0 (0.0%)	10 (13.0%)	4 (5.2%)	1 (1.3%)	2 (2.6%)	18 (23.4%)	28 (36.4%)	14 (18.2%)	<0.001
Negative	209	19 (9.1%)	81 (38.8%)	36 (17.2%)	15 (7.2%)	14 (6.7%)	16 (7.7%)	13 (6.2%)	15 (7.2%)	
Benzodiazepine										
Positive	159	12 (7.5%)	55 (34.6%)	25 (15.7%)	5 (3.1%)	2 (1.3%)	15 (9.4%)	22 (13.8%)	23 (14.5%)	<0.001
Negative	127	7 (5.5%)	36 (28.3%)	15 (11.8%)	11 (8.7%)	14 (11.0%)	19 (15.0%)	19 (15.0%)	6 (4.7%)	
Cocaine										
Positive	135	10 (7.4%)	51 (37.8%)	28 (20.7%)	9 (6.7%)	6 (4.4%)	4 (3.0%)	18 (13.3%)	9 (6.7%)	<0.001
Negative	151	9 (6.0%)	40 (26.5%)	12 (7.9%)	7 (4.6%)	10 (6.6%)	30 (19.9%)	23 (15.2%)	20 (13.2%)	
THC/Cannabis										
Positive	144	8 (5.6%)	40 (27.8%)	23 (16.0%)	5 (3.5%)	7 (4.9%)	20 (13.9%)	25 (17.4%)	16 (11.1%)	0.288
Negative	142	11 (7.7%)	51 (35.9%)	17 (12.0%)	11 (7.7%)	9 (6.3%)	14 (9.9%)	16 (11.3%)	13 (9.2%)	
Opiate										
Positive	177	6 (3.4%)	56 (31.6%)	33 (18.6%)	8 (4.5%)	7 (4.0%)	21 (11.9%)	26 (14.7%)	20 (11.3%)	0.010
Negative	109	13 (11.9%)	35 (32.1%)	7 (6.4%)	8 (7.3%)	9 (8.3%)	13 (11.9%)	15 (13.8%)	9 (8.3%)	
Oxycodone										
Positive	89	6 (6.7%)	20 (22.5%)	22 (24.7%)	2 (2.2%)	3 (3.4%)	14 (15.7%)	10 (11.2%)	12 (13.5%)	0.003
Negative	197	13 (6.6%)	71 (36.0%)	18 (9.1%)	14 (7.1%)	13 (6.6%)	20 (10.2%)	31 (15.7%)	17 (8.6%)	

Supplementary Table S2

Baseline Characteristics of the Study Participants From CHC Sites

Characteristic	Total (N=443)	CHC 1 (N=53)	CHC 2 (N=16)	CHC 3 (N=26)	CHC 4 (N=87)	CHC 5 (N=72)	CHC 6 (N=44)	CHC 7 (N=85)	CHC 8 (N=60)	p
**Gender**										0.251
Female	120	13 (10.8%)	8 (6.7%)	5 (4.2%)	22 (18.3%)	26 (21.7%)	15 (12.5%)	18 (15.0%)	13 (10.8%)	
Male	318	40 (12.6%)	8 (2.5%)	21 (6.6%)	65 (20.4%)	46 (14.5%)	28 (8.8%)	65 (20.4%)	45 (14.2%)	
Transgender or Gender Non-conforming	4	0 (0.0%)	0 (0.0%)	0 (0.0%)	0 (0.0%)	0 (0.0%)	1 (25.0%)	2 (50.0%)	1 (25.0%)	
**Age [M (SD)]**	43.8 (11.7)	38.2 (10.5)	45.3 (12.6)	48.3 (10.6)	47.8 (10.4)	35.8 (8.5)	42.2 (11.8)	48.4 (11.5)	44.8 (11.7)	<0.001
**Race**										<0.001
White/Caucasian	279	46 (16.5%)	10 (3.6%)	6 (2.2%)	33 (11.8%)	70 (25.1%)	21 (7.5%)	50 (17.9%)	43 (15.4%)	
Black/African American	78	2 (2.6%)	1 (1.3%)	2 (2.6%)	46 (59.0%)	0 (0.0%)	2 (2.6%)	16 (20.5%)	9 (11.5%)	
Other	77	5 (6.5%)	5 (6.5%)	18 (23.4%)	8 (10.4%)	2 (2.6%)	12 (15.6%)	19 (24.7%)	8 (10.4%)	
**Latino/Hispanic Ethnicity**										<0.001
No	369	50 (13.6%)	12 (3.3%)	8 (2.2%)	86 (23.3%)	72 (19.5%)	13 (3.5%)	73 (19.8%)	55 (14.9%)	
Yes	74	3 (4.1%)	4 (5.4%)	18 (24.3%)	1 (1.4%)	0 (0.0%)	31 (41.9%)	12 (16.2%)	5 (6.8%)	
**Marital/cohabitation Status**										0.122
Single/Separated /Divorced/Widowed	380	38 (10.0%)	14 (3.7%)	18 (4.7%)	78 (20.5%)	62 (16.3%)	40 (10.5%)	76 (20.0%)	54 (14.2%)	
Married/living together as married	44	3 (6.8%)	1 (2.3%)	8 (18.2%)	8 (18.2%)	8 (18.2%)	3 (6.8%)	8 (18.2%)	5 (11.4%)	
Other	5	1 (20.0%)	1 (20.0%)	0 (0.0%)	0 (0.0%)	0 (0.0%)	1 (20.0%)	1 (20.0%)	1 (20.0%)	
**Education**										<0.001
Less than HS	89	6 (6.7%)	11 (12.4%)	8 (9.0%)	21 (23.6%)	10 (11.2%)	8 (9.0%)	14 (15.7%)	11 (12.4%)	
HS diploma or GED	177	12 (6.8%)	3 (1.7%)	11 (6.2%)	45 (25.4%)	37 (20.9%)	16 (9.0%)	30 (16.9%)	23 (13.0%)	
Some college or more	163	24 (14.7%)	2 (1.2%)	7 (4.3%)	20 (12.3%)	23 (14.1%)	20 (12.3%)	41 (25.2%)	26 (16.0%)	
**Living stability**										<0.001
Stable housing	205	10 (4.9%)	8 (3.9%)	16 (7.8%)	27 (13.2%)	33 (16.1%)	17 (8.3%)	59 (28.8%)	35 (17.1%)	
Unstable housing	224	32 (14.3%)	8 (3.6%)	10 (4.5%)	59 (26.3%)	37 (16.5%)	27 (12.1%)	26 (11.6%)	25 (11.2%)	
**Availability of transportation**										<0.001
Yes	173	26 (15.0%)	5 (2.9%)	4 (2.3%)	16 (9.2%)	43 (24.9%)	16 (9.2%)	48 (27.7%)	15 (8.7%)	
Maybe, if I can get a ride	27	2 (7.4%)	2 (7.4%)	0 (0.0%)	5 (18.5%)	11 (40.7%)	6 (22.2%)	1 (3.7%)	0 (0.0%)	
Maybe, if public transportation is available	224	14 (6.3%)	9 (4.0%)	22 (9.8%)	64 (28.6%)	12 (5.4%)	22 (9.8%)	36 (16.1%)	45 (20.1%)	
No	6	1 (16.7%)	0 (0.0%)	0 (0.0%)	1 (16.7%)	4 (66.7%)	0 (0.0%)	0 (0.0%)	0 (0.0%)	
**Employed with a regular job or informal work**										<0.001
Yes	141	22 (15.6%)	4 (2.8%)	3 (2.1%)	24 (17.0%)	40 (28.4%)	20 (14.2%)	14 (9.9%)	14 (9.9%)	
No	288	20 (6.9%)	12 (4.2%)	23 (8.0%)	62 (21.5%)	30 (10.4%)	24 (8.3%)	71 (24.7%)	46 (16.0%)	
**Medication for OUD**										<0.001
None	191	16 (8.4%)	0 (0.0%)	17 (8.9%)	17 (8.9%)	38 (19.9%)	20 (10.5%)	46 (24.1%)	37 (19.4%)	
Buprenorphine only	102	18 (17.6%)	4 (3.9%)	0 (0.0%)	28 (27.5%)	29 (28.4%)	7 (6.9%)	6 (5.9%)	10 (9.8%)	
Methadone	123	7 (5.7%)	12 (9.8%)	6 (4.9%)	39 (31.7%)	4 (3.3%)	12 (9.8%)	32 (26.0%)	11 (8.9%)	
**Depressive symptoms (PHQ-9) [M (SD)]**	10.1 (6.2)	8.9 (5.3)	11.1 (5.8)	11.6 (5.9)	8.7 (5.9)	11.7 (6.9)	9.9 (5.4)	10.2 (6.3)	10.5 (6.4)	0.081
**Anxiety symptoms (GAD-7) [M (SD)]**	8.7 (6.1)	7.5 (6.3)	9.2 (4.9)	9.9 (5.1)	7.7 (5.8)	10.7 (6.7)	8.7 (5.1)	7.9 (6.0)	9.4 (6.4)	0.066

**Previously Received HCV Treatment (non-DAA)**										0.192
No	429	51 (11.9%)	15 (3.5%)	25 (5.8%)	87 (20.3%)	70 (16.3%)	42 (9.8%)	79 (18.4%)	60 (14.0%)	
Yes	14	2 (14.3%)	1 (7.1%)	1 (7.1%)	0 (0.0%)	2 (14.3%)	2 (14.3%)	6 (42.9%)	0 (0.0%)	
**Cirrhosis**										0.156
No	409	46 (11.2%)	14 (3.4%)	22 (5.4%)	80 (19.6%)	68 (16.6%)	40 (9.8%)	79 (19.3%)	60 (14.7%)	
Yes	34	7 (20.6%)	2 (5.9%)	4 (11.8%)	7 (20.6%)	4 (11.8%)	4 (11.8%)	6 (17.6%)	0 (0.0%)	
**HIV infection (positive)**										<0.001
No	239	41 (17.2%)	12 (5.0%)	17 (7.1%)	52 (21.8%)	62 (25.9%)	21 (8.8%)	1 (0.4%)	33 (13.8%)	
Yes	89	6 (6.7%)	4 (4.5%)	1 (1.1%)	18 (20.2%)	0 (0.0%)	8 (9.0%)	26 (29.2%)	26 (29.2%)	
**Alcohol misuse**										0.754
No	255	27 (10.6%)	10 (3.9%)	12 (4.7%)	48 (18.8%)	44 (17.3%)	21 (8.2%)	55 (21.6%)	38 (14.9%)	
Yes	156	14 (9.0%)	6 (3.8%)	11 (7.1%)	36 (23.1%)	26 (16.7%)	17 (10.9%)	27 (17.3%)	19 (12.2%)	
**Last drug injection (within 3 months of screening)**										0.002
0-4 weeks	337	36 (10.7%)	11 (3.3%)	20 (5.9%)	58 (17.2%)	56 (16.6%)	29 (8.6%)	79 (23.4%)	48 (14.2%)	
5-8 weeks	67	11 (16.4%)	5 (7.5%)	4 (6.0%)	21 (31.3%)	8 (11.9%)	8 (11.9%)	1 (1.5%)	9 (13.4%)	
9-12 weeks	38	6 (15.8%)	0 (0.0%)	2 (5.3%)	8 (21.1%)	8 (21.1%)	7 (18.4%)	4 (10.5%)	3 (7.9%)	
**Number of days injected drugs in the past 3 months [M (SD)]**	37.1 (32.7)	21.3 (22.8)	34.7 (34.8)	40.7 (35.4)	34.9 (30.1)	51.0 (37.2)	30.0 (32.0)	37.9 (31.3)	37.3 (32.3)	0.005
**Times injecting drugs a day [M (SD)]**	3.4 (3.1)	4.7 (3.8)	2.8 (2.4)	4.8 (6.1)	3.7 (3.4)	4.4 (3.6)	2.8 (1.5)	2.1 (1.2)	2.7 (2.0)	<0.001
**Urine drug screen results at baseline visit:**										
Any drug										
No	25	5 (20.0%)	0 (0.0%)	0 (0.0%)	4 (16.0%)	8 (32.0%)	4 (16.0%)	3 (12.0%)	1 (4.0%)	0.074
Yes	371	34 (9.2%)	16 (4.3%)	21 (5.7%)	70 (18.9%)	64 (17.3%)	30 (8.1%)	81 (21.8%)	55 (14.8%)	
Amphetamine										
Positive	130	8 (6.2%)	1 (0.8%)	0 (0.0%)	2 (1.5%)	16 (12.3%)	20 (15.4%)	51 (39.2%)	32 (24.6%)	<0.001
Negative	266	31 (11.7%)	15 (5.6%)	21 (7.9%)	72 (27.1%)	56 (21.1%)	14 (5.3%)	33 (12.4%)	24 (9.0%)	
Methamphetamine										
Positive	141	2 (1.4%)	0 (0.0%)	1 (0.7%)	8 (5.7%)	17 (12.1%)	19 (13.5%)	59 (41.8%)	35 (24.8%)	<0.001
Negative	255	37 (14.5%)	16 (6.3%)	20 (7.8%)	66 (25.9%)	55 (21.6%)	15 (5.9%)	25 (9.8%)	21 (8.2%)	
Benzodiazepine										
Positive	199	20 (10.1%)	13 (6.5%)	17 (8.5%)	33 (16.6%)	23 (11.6%)	16 (8.0%)	40 (20.1%)	37 (18.6%)	<0.001
Negative	197	19 (9.6%)	3 (1.5%)	4 (2.0%)	41 (20.8%)	49 (24.9%)	18 (9.1%)	44 (22.3%)	19 (9.6%)	
Cocaine										
Positive	152	9 (5.9%)	13 (8.6%)	13 (8.6%)	38 (25.0%)	16 (10.5%)	7 (4.6%)	34 (22.4%)	22 (14.5%)	<0.001
Negative	243	29 (11.9%)	3 (1.2%)	8 (3.3%)	36 (14.8%)	56 (23.0%)	27 (11.1%)	50 (20.6%)	34 (14.0%)	
THC/Cannabis										
Positive	193	14 (7.3%)	9 (4.7%)	6 (3.1%)	28 (14.5%)	27 (14.0%)	18 (9.3%)	52 (26.9%)	39 (20.2%)	<0.001
Negative	203	25 (12.3%)	7 (3.4%)	15 (7.4%)	46 (22.7%)	45 (22.2%)	16 (7.9%)	32 (15.8%)	17 (8.4%)	
Opiate										
Positive	173	6 (3.5%)	10 (5.8%)	13 (7.5%)	29 (16.8%)	21 (12.1%)	18 (10.4%)	44 (25.4%)	32 (18.5%)	<0.001
Negative	223	33 (14.8%)	6 (2.7%)	8 (3.6%)	45 (20.2%)	51 (22.9%)	16 (7.2%)	40 (17.9%)	24 (10.8%)	
Oxycodone										
Positive	93	3 (3.2%)	5 (5.4%)	8 (8.6%)	8 (8.6%)	11 (11.8%)	10 (10.8%)	28 (30.1%)	20 (21.5%)	<0.001
Negative	303	36 (11.9%)	11 (3.6%)	13 (4.3%)	66 (21.8%)	61 (20.1%)	24 (7.9%)	56 (18.5%)	36 (11.9%)	
